# Fetal Hemoglobin in Preterm Infants After Resuscitation with Immediate Cord Clamping, Delayed Cord Clamping, or Cord Milking

**DOI:** 10.3390/children12050627

**Published:** 2025-05-13

**Authors:** Carlo Dani, Giulia Remaschi, Matilde Ulivi, Niccolò Monti, Simone Pratesi

**Affiliations:** 1 Department of Neurosciences, Psychology, Drug Research and Child Health, University of Florence, 50139 Florence, Italy; matilde.ulivi1@stud.unifi.it (M.U.); nicolo.monti@stud.unifi.it (N.M.); simone.pratesi@unifi.it (S.P.); 2 Division of Neonatology, Careggi University Hospital of Florence, Largo Brambilla, 3, 50141 Florence, Italy; remaschig@aou-careggi.toscana.it

**Keywords:** fetal hemoglobin, delayed cord clamping, umbilical cord milking, preterm infant

## Abstract

**Background:** Fetal hemoglobin (HbF) plays a beneficial role in the progressive adaptation to the postnatal oxygen-rich environment in preterm infants due to its peculiar properties. Our aim was to evaluate if preterm infants resuscitated with delayed cord clamping (DCC) or umbilical cord milking (UCM) might have higher and more durable HbF levels than infants resuscitated with immediate cord clamping (ICC). **Methods:** We retrospectively studied 181 preterm infants born at <30 weeks of gestation, among whom 120 were resuscitated with ICC, 30 with DCC, and 31 with UCM. Mean values of HbF blood levels in the first postnatal week (HbF1st _week_); in the 14th, 21st, and 28th days of life (HbF_14–21–28 DOL_); and in the 31st, 34th, and 36th weeks of postmenstrual age (HbF_31–34–36 weeks_) were calculated. **Results:** We found that HbF1st _week_ (15.3 ± 3.4 vs. 12.6 ± 3.5 g/dL, *p* < 0.001), HbF_14–21–28 DOL_, (9.3 ± 3.2 vs. 7.6 ± 3.6 g/dL, *p* = 0.018), and Hb_–34–36 weeks_ (7.5 ± 3.6 vs. 5.7 ± 3.6 g/dL, *p* = 0.014) levels were higher in the UCM than in the ICC group. No differences of HbF levels were found between the DCC and ICC groups. **Conclusions:** UCM was associated with a persistent higher level of HbF than ICC. The effect of DCC was less marked as HbF level was higher than ICC only in the first week of life. UCM and DCC may help counteract the negative effects of blood sampling and transfusions on HbF levels.

## 1. Introduction

Fetal hemoglobin (HbF) and adult hemoglobin (HbA) have a similar structure but different properties that confer HbF with a greater affinity for oxygen and a greater capacity to deliver oxygen to tissues [1]. HbF constitutes 70–90% of total hemoglobin in fetuses and is optimal for their development in the hypoxic uterine environment [2]. Even in preterm infants, HbF constitutes 90% of total hemoglobin at birth [1], but it dramatically decreases during the first fifteen days due to blood samplings, which cause iatrogenic blood loss of up to 58% of circulating blood volume [3]. Therefore, preterm infants require frequent transfusions of adult blood, and HbA replaces HbF. Unfortunately, the reduced oxygen affinity of HbA increases oxygen delivery to the tissues and may promote hyperoxia and oxidative stress [4] in patients with an immature enzymatic antioxidant system [5]. HbF also has direct antioxidant effects, such as a pseudo-peroxidase activity that neutralizes peroxides and scavenges free radicals [6,7].

Therefore, the postnatal decrease of HbF and its replacement with transfused HbA have the combined adverse effects of increasing tissue hyperoxia and decreasing antioxidant defenses in preterm infants. This explains why a low fraction of HbF in the blood was associated with a higher risk of developing complications of prematurity related to oxidative stress, such as retinopathy of prematurity (ROP) [1,8,9] and bronchopulmonary dysplasia (BPD) [10].

Resuscitation with delayed cord clamping (DCC) for 30–60 s improves the outcomes of preterm infants who do not require immediate resuscitation at birth compared to ICC, as it allows smoother cardiorespiratory adaptation [11,12,13]. Umbilical cord milking (UCM) still represents a procedure that can be performed at birth when DCC is not possible (i.e., if immediate resuscitation is required), since it provides greater advantages to preterm infants than ICC [13,14,15,16,17].

A mechanism by which DCC and UCM may improve preterm infants’ outcome is the increase in blood volume due to placental blood transfusion. Resuscitation DCC for 30–120 s was found to increase blood volume by 18% in comparison with ICC in preterm infants [18]. A single UCM was found to provide preterm infants with approximately 18 g/dL of additional blood volume [19]. Moreover, milking the cord four times achieved a similar amount of placental blood transfusion compared with DCC for 30 s [20]. An additional benefit of DCC and UCM in preterm infants may be the postnatal increase in HbF levels and the longer persistence of its protective effects. However, to date, no data are available on the effects of these different cord blood management strategies on postnatal blood HbF levels.

On this basis, we hypothesized that preterm infants resuscitated with DCC or UCM might have persistently higher HbF levels than infants resuscitated with ICC. To assess this hypothesis, we performed this retrospective study in preterm infants in which the association between HbF levels and fractions, and different strategies of cord management, was evaluated.

## 2. Material and Methods

This is a retrospective observational study that was carried out in a cohort of preterm infants born at <30 weeks of gestation. They were born from November 2017 to June 2024 in the neonatal intensive care unit (NICU) of the Careggi University Hospital of Florence. The study was approved by the Tuscany pediatric ethics committee. Patients who received resuscitation with DCC or UCM were previously included in the multicenter PCI trial [21]. Exclusion criteria were placental and cord abnormalities, major congenital malformations and metabolic disorders, genetic syndromes, fetal hydrops, and death within the first seven days of life.

### 2.1. HbF Blood Levels and Fractions

Arterial or capillary blood gas analyses reported in the electronic medical records of each infant were reviewed. HbF levels and fractions were averaged for each day in the first week of life, and subsequently on days 14, 21, and 28 of life, and on weeks 31, 34, and 36 of postmenstrual age. In agreement with previous studies [8,10], mean values of the first postnatal week (HbF1st _week_); of 14, 21 and 28 days of life (HbF_14–21–28 DOL_); and 31, 34 and 36 weeks of postmenstrual age (HbF_31–34–36 weeks_) were evaluated in the data analysis as a representation of these variables, since they were based on similarly distributed pooled values and allowed for increased precision of the data while limiting variability.

### 2.2. Resuscitation and Cord Clamping

Patients were resuscitated following current guidelines of the American Academy of Pediatrics [22,23]. In the UCM group, 20 cm of the intact umbilical cord was squeezed over 2 s, repeated for a total of 4 times, and then the cord was clamped and cut within 20 s of life. In the DCC group, the umbilical cord was not milked and was clamped 180 s after birth.

### 2.3. Data Collection

The following data were reported for the infants studied: gestational age; birth weight; sex; antenatal steroids; type of delivery; resuscitation with ICC, DCC, or UCM; clinical chorioamnionitis; Apgar score at 5 min; noninvasive and invasive respiratory support; diagnosis of sepsis; BPD; IVH; ROP; necrotizing enterocolitis (NEC) > 2 grade; duration of hospitalization; and death.

Sepsis was diagnosed by at least one positive blood or cerebrospinal fluid culture. BPD was defined using the classification of Jobe et al. [24], as was IVH using the classification of Papile et al. [25], NEC using the Bell’s criteria [26], and ROP using the International Classification of ROP [27].

Blood HbF levels and fractions were measured using a blood gas analyzer (ABL800, RadiometerMedical ApS, Brønshøj, Copenhagen, Denmark). HbF levels were reported as g/dL and as a percentage of the total hemoglobin (Hb).

The decision to perform red blood cell transfusions was taken in accordance with the guidelines of the Italian Society of Neonatology [28].

### 2.4. Primary and Secondary Endpoints

The primary endpoint of our study was to evaluate the possible differences in HbF blood level in infants resuscitated with UCM versus ICC in the first weeks of life (HbF1st _week_). Secondary endpoints were the evaluation of possible differences in HbF blood level in infants resuscitated with DCC versus ICC in the first weeks of life (HbF1st _week_), and HbF blood fractions in infants resuscitated with UMC, DCC, or ICC in the first weeks of life (HbF1st _week_). Other secondary endpoints were the comparison of HbF levels and fractions measured at subsequent datapoints (HbF_14–21–28 DOL_, HbF_31–34–36 weeks_) in infants resuscitated with ICC, DCC, or UCM.

### 2.5. Statistical Analysis

We have previously calculated in our population that the mean value of HbF1st _week_ is 12.6 ± 3.5 g/dL in infants resuscitated with ICC. Therefore, we calculated that a sample size of at least 30 infants in each group was needed to detect as statistically significant a 20% difference in HbF1st _week_ between infants resuscitated with UCM versus those resuscitated with ICC, with a 80% power at the 0.05 level.

The clinical characteristics of infants were reported as mean and standard deviation, mean frequency, or median and interquartile range (IQR). Missing data were replaced with an estimated value based on available information by mean imputation. Comparisons between the groups were performed using the Student’s *t*-test for parametric continuous variables, the two sample Wilcoxon rank-sum test for non-parametric continuous variables, and the Χ^2^ test for categorical variables. To test the normality of our data, the Shapiro–Wilk test was used. The intragroup variation in HbF blood levels and fractions was compared by repeated-measures analysis of variance (ANOVA). A *p* value of <0.05 was considered statistically significant.

## 3. Results

We studied 181 preterm infants, of whom 120 were resuscitated with ICC, 30 with DCC, and 31 with UCM. Their demographic and clinical characteristics were similar and are shown in Table 1.

We found that Hb blood level decreased from 16.3 ± 2.5 g/dL on day 1 day to 9.9 ± 1.2 g/dL (*p* < 0.001) in the 36th week of life the ICC group, from 17.2 ± 3.1 g/dL to 10.0 ± 1.0 g/dL (*p* < 0.001) in the DCC group, and from 17.7 ± 2.3 g/dL to 10.3 ± 1.8 g/dL (*p* < 0.001) in the UCM group. The HbF1st _week_ level was higher in the UCM than in the ICC group, while Hb_14–21–28 DOL_ and Hb_31–34–36 weeks_ levels were similar between the groups (Table S1).

### HbF Levels and Fractions

HbF1st _week_, HbF_14–21–28 DOL_, and HbF_31–34–36 weeks_ levels and fractions progressively decreased (*p* < 0.001) in all the groups (Table 2).

**Figure 1:**
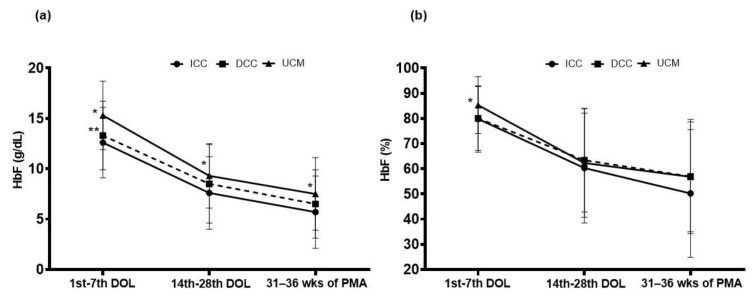
Changes of HbF (**a**) volume and (**b**) fractions measured during the first week of life (1st–7th days of life, DOL); on days 14, 21, and 28 of life (14th–28th DOL); and on weeks 31, 34, and 36 of postmenstrual age (31–36 weeks of PMA) in infants resuscitated with immediate cord clamping (ICC), delayed cord clamping (DCC), or umbilical cord milking (UCM). Means and SDs. * *p* < 0.05 vs. ICC; ** *p* < 0.05 vs. ICC.

We found that HbF1st _week_ (15.3 ± 3.4 vs. 12.6 ± 3.5 g/dL, *p* < 0.001), HbF_14–21–28 DOL_ (9.3 ± 3.2 vs. 7.6 ± 3.6 g/dL, *p* = 0.018), and HbF_31–34–36 weeks_ (7.5 ± 3.6 vs. 5.7 ± 3.6 g/dL, *p* = 0.014) levels were higher in the UCM than in the ICC group. HbF1st _week_ (15.3 ± 3.4 vs. 13.3 ± 4.0 g/dL, *p* < 0.001) was higher in the UCM than in the DCC group. No differences of HbF levels were found between DCC and ICC groups (Table 2, Figure 1).

HbF1st _week_ fraction (85.3 ± 11.3 vs. 79.8 ± 13.2%, *p* = 0.035) was higher in the UCM than in the ICC group. No differences in HbF fractions were found between DCC and UCM groups (Table 2, Figure 1).

Daily and weekly changes in Hb levels and HbF levels and fractions are detailed in Tables S1–S3.

## 4. Discussion

In this study, we evaluated for the first time the effect of DCC and UCM in comparison with ICC on levels and fractions of HbF in very preterm infants. We found that UCM was associated with a higher HbF volume than ICC and that this effect persisted until the 36th week of postmenstrual age. The effect of DCC, however, was less marked since HbF volume was significantly higher than that of ICC only in the first week of life, while in the subsequent study period it showed only an increasing trend. These results demonstrated that partial placental blood transfusion induced by UCM and DCC is associated with the beneficial increase in blood HbF level in very preterm infants.

We could not compare our results with previous ones since this topic has not been addressed before. Previous studies were focused on the increase of circulating blood volume induced by DCC and UCM in preterm infants in comparison with ICC but did not investigate changes in HbF levels and fractions. However, they provide useful information for the interpretation of our results. Aladangady et al. demonstrated that DCC significantly increased mean blood volume in preterm infants ≤33 weeks of gestational age compared with ICC (74.4 vs. 62.7 g/dL) [18]. Furthermore, they found that delaying cord clamping from 60 to 90 s did not lead to further increases in blood volume [18]. Hosono et al. reported that a single 30 cm UCM of umbilical cord could transfuse approximately 18 g/dL of placental blood in extremely preterm infants [19]. Chaowawanit et al. calculated that the umbilical cord in very-low-birth-weight infants contains approximately 0.41 mL per cm, suggesting that birth weight and umbilical cord length should be considered when assessing the appropriate blood volume for a preterm infant [29]. Rabe et al. found that a 30 s DCC achieved a similar amount of placental blood transfusion compared to four times UCM [12]. Therefore, the increase in Hb levels and, consequently, in HbF levels that we found in our patients resuscitated with DCC or UCM is consistent with previous studies [18,19,20], since partial differences between our patients resuscitated with DCC and UCM [20] could depend on the modalities of performance of the procedures (e.g., duration of DCC, number of umbilical cord milkings, and length of umbilical cord milked) [12,18,19,29].

We found that UCM, and to lesser extent DCC, increased the blood level of HbF in comparison with ICC, not only in the first weeks of life but also later, when the protective effect of HbF against oxidative stress [4,5,6,7] continues to be important in very preterm infants to prevent complications. Previous studies on HbF synthesis in preterm and term human infants demonstrated that up to the postconceptional age of 37 weeks, HbF remains the major hemoglobin synthesized [30,31]. A decrease in HbF fraction from approximately 80–90% at gestational age <32 weeks to 50–60% at 38–42 weeks of gestation was observed [30]. Interestingly, the rate of transition from HbF to HbA synthesis in the postnatal period in preterm infants reflects that which occurs during intrauterine life, since birth does not appear to accelerate the transition to HbA synthesis [30]. Once term gestational age is reached, the decline in HbF synthesis is relatively rapid, with the HbF fraction decreasing to approximately 30% at 8 postnatal weeks, to 3.6% at 28 weeks, and to only 0.8% at 49 postnatal weeks [31]. Therefore, any procedure that helps maintain high levels of HbF in preterm infants is consistent with what happens physiologically during fetal life and effectively helps counteract the negative effects of blood sampling and subsequent transfusions that lower the level of HbF by replacing it with HbA.

The cord management is a crucial aspect of the resuscitation of preterm infants in the delivery room, and the knowledge of the beneficial effect of UCM and DCC on HbF values in the neonatal period is consistent with growing evidence on the importance of maintaining high HbF levels to prevent prematurity complications and improve outcomes in preterm infants [1,8,9,10]. There is a rising interest in the protective role of HbF and in the development of new strategies to maintain its blood levels in preterm infants. Red blood cell transfusion using allogeneic cord blood collected from term deliveries containing HbF-rich red blood cells [32] and effective postnatal cord management are included in these strategies.

The limitations of our study include its retrospective design, which prevented the possibility of recording some important data, such blood volume loss due to phlebotomy. However, the data presented reflect care provided in a single center in a homogeneous population, and statistical analysis was accurate. This suggests that our findings are correct and reliable. The main strength of this study is the novelty of the topic and the fact that it detailed the changes in blood levels and fractions of HbF over a long period of time.

## 5. Conclusions

We demonstrated that UCM was associated with a persistently higher HbF volume than ICC, while the effect of DCC was less marked, since HbF volume was higher than ICC only in the first week of life. UCM and DCC may help counteract the negative effects of blood sampling and RBC transfusions on HbF levels and improve the prognosis of preterm infants, hopefully helping to decrease the risk of ROP and BPD, in part by maintaining high HbF levels.

## Figures and Tables

**Table 1:** Demographic and clinical data in infants who were resuscitated with immediate cord clamping (ICC), delayed cord clamping (DCC), or umbilical cord milking (UCM). Mean (±SD) or rate (%) or median and interquartile range (IQR).

	ICC (n = 120)	DCC (n = 30)	UCM (n = 31)
Gestational age (wks)	27.0 ± 1.6	26.6 ± 1.4	27.3 ± 1.5
Birth weight (g)	974 ± 266	922 ± 197	976 ± 288
Female	67 (59)	12 (40)	14 (45)
Antenatal steroids	108 (90)	30 (100)	31 (100)
Cesarean section	76 (63)	15 (50)	18 (58)
Chorioamnionitis	4 (3)	4 (13)	2 (6)
Apgar score at 5 min	8 (7–8)	8 (8–8)	8 (7–8)
Noninvasive ventilation Duration (d)	119 (99) 26 ± 11	28 (93) 26 ± 19	30 (100) 26 ± 18
Mechanical ventilation Duration (d)	50 (42) 7 ± 14	17 (57) 11 ± 15	9 (29) 7 ± 18
Sepsis	41 (34)	12 (40)	11 (35)
BPD	79 (66)	23 (77)	19 (61)
Intraventricular hemorrhage	28 (23)	9 (30)	9 (29)
Retinopathy of prematurity	26 (22)	4 (13)	4 (13)
Necrotizing enterocolitis	13 (11)	3 (10)	0
Death	2 (2)	3 (10)	1 (3)
Duration of hospital stay (d)	87 ± 34	86 ± 30	79 ± 33
HbF level (g/dL) HbF fraction nadir level (%) Age at lowest level (d)	10.3 ± 1.5 31.0 ± 20.3 43.0 ± 15.5	11.0 ± 1.5 33.1 ± 16.2 39.2 ± 18.8	11.0 ± 1.9 37.1 ± 21.6 36.1 ± 15.6
Patients transfused with RBCs transfusions Number of transfusions	74 (62) 1 (0–3)	15 (50) 1 (0–2)	15 (48) 1 (0–2)

DOL: day of life; PMA: postmenstrual age; and RBCs: red blood cells.

**Table 2:** Mean values blood levels (g/dL) and fractions (%) of fetal hemoglobin (HbF), and blood levels (g/dL) of hemoglobin (Hb) measured between 1 and 7 days of life (HbF1st _week_), at 14, 21, and 28 days of life (HbF_14–21–28 DOL_), and at 31, 34, and 36 weeks of postmenstrual age (HbF_31–34–36 weeks_) in infants who were resuscitated with immediate (ICC) or delayed cord clamping (DCC) or umbilical cord milking (UCM). Mean ± SD.

	ICC (n = 120)	DCC (n = 30)	UCM (n = 31)	*p* ICC vs. UCM
**HbF (g/dL)**				
HbF1st _week_	12.6 ± 3.5	13.3 ± 4.0 *	15.3 ± 3.4	<0.001
HbF_14–21–28 DOL_	7.6 ± 3.6	8.5 ± 3.9	9.3 ± 3.2	0.018
HbF_31–34–36 weeks_	5.7 ± 3.6	6.5 ± 3.4	7.5 ± 3.6	0.014
*p*	<0.001	<0.001	<0.001	
**HbF (%)**				
HbF1st _week_	79.8 ± 13.2	80.0 ± 12.7	85.3 ± 11.3	0.035
HbF_14–21–28 DOL_	60.3 ± 21.8	63.4 ± 20.6	62.3 ± 21.6	0.650
HbF_31–34–36 weeks_	50.2 ± 25.4	56.9 ± 22.7	56.8 ± 21.8	0.187
*p*	<0.001	<0.001	<0.001	
**Hb (g/dL)**				
Hb1st _week_	15.6 ± 2.6	16.3 ± 3.0	17.9 ± 2.5	<0.001
Hb_14–21–28 DOL_	12.2 ± 2.3	12.8 ± 2.5	12.6 ± 2.3	0.389
Hb_31–34–36 weeks_	10.9 ± 2.1	11.0 ± 2.1	11.2 ± 2.6	0.502
*p*	<0.001	<0.001	<0.001	

* *p* = 0.039 UCM vs. DCC. DOL: day of life. PMA: postmenstrual age.

## Data Availability

All relevant data are within the manuscript.

## Supplementary Materials

The following supporting information can be downloaded at: https://www.mdpi.com/article/10.3390/children12050627/s1, Table S1. Hemoglobin (Hb) level (mg/dL) measured every day between 1 and 7 days of life, at 14, 21, and 28 day of life and at 31, 34, and 36 weeks of postmenstrual age in infants who had resuscitation with immediate cord clamping (ICC), delayed cord clamping (DCC) or umbilical cord milking (UCM). Mean ± SD. Table S2. Fetal hemoglobin (HbF) blood fractions (%) measured every day between 1 and 7 days of life, at 14, 21, and 28 day of life and at 31, 34, and 36 weeks of postmenstrual age in infants who had resuscitation with immediate cord clamping (ICC), delayed cord clamping (DCC) or umbilical cord milking (UCM). Mean ± SD. Table S3. Fetal hemoglobin (HbF) levels (g/dL) measured between 1 and 7 days of life, at 14, 21, and 28 day of life and at 31, 34, and 36 weeks of postmenstrual age in infants who had resuscitation with immediate cord clamping (ICC), delayed cord clamping (DCC) or umbilical cord milking (UCM). Mean ± SD.

## Abbreviations

HbF	fetal hemoglobin
HbF1st _week_	mean values of HbF volume or fractions during the first week of life
HbF_14–21–28 DOL_	mean values of HbF volume or fractions on the 14th, 21st, and 28th days of life
HbF_31–36 weeks_	mean values of HbF fractions during the 31st, 34th, and 36th weeks of postmenstrual age
