# Supplementary material for: Fetal Hemoglobin in Preterm Infants After Resuscitation with Immediate Cord Clamping, Delayed Cord Clamping, or Cord Milking
Source: Children (Basel). 2025 May 13;12(5):627. doi: 10.3390/children12050627 (PMC12110533; doi:10.3390/children12050627)
Supplement: Supplementary file 1 [file children-12-00627-s001.zip › children-3579923-supplementary.pdf]

Table S1. Hemoglobin (Hb) level (mg/dL) measured every day between 1 and 7 days of life, at 14, 21, and 28 day of life and at 31, 34, and 36 weeks of postmenstrual age in infants who had resuscitation with immediate cord clamping (ICC), delayed cord clamping (DCC) or umbilical cord milking (UCM). Mean±SD.

	**ICC (n=120)**	**DCC (n=30)**	**UCM (n=31)**
1st DOL	16.3±2.5	17.2±3.1	17.7±2.3*
2nd DOL	16.5±2.6	17.0±2.7	17.4±2.5
3rd DOL	15.8±2.5	16.4±2.9	16.3±2.3
4th DOL	15.4±2.5	16.0±3.1	16.3±2.1
5th DOL	15.1±2.6	16.1±3.1	16.0±2.5
6th DOL	15.0±2.4	15.8±3.0	15.7±2.5
7th DOL	14.8±2.3	15.4±3.4	15.5±2.8
14th DOL	13.1±2.3	13.8±2.7	13.6±3.0
21st DOL	12.2±2.4	12.7±2.5	12.5±1.7
28th DOL	11.3±2.5	12.0±2.1	11.7±1.5
31st week of PMA	12.2±2.5	12.2±2.3	12.7±3.2
34th week of PMA	10.4±1.8	10.0±1.4	10.4±1.6
36th weeks of PMA	9.9±1.2	10.0±1.0	10.3±1.8
P	<0.001	<0.001	<0.001

*P=0.005 vs. ICC

DOL: day of life PMA: postmenstrual age

Table S2. Fetal hemoglobin (HbF) blood fractions (%) measured every day between 1 and 7 days of life, at 14, 21, and 28 day of life and at 31, 34, and 36 weeks of postmenstrual age in infants who had resuscitation with immediate cord clamping (ICC), delayed cord clamping (DCC) or umbilical cord milking (UCM). Mean±SD.

	**ICC (n=120)**	**DCC (n=30)**	**UCM (n=31)**
1st DOL	84.5±9.4	84.3±8.8	84.5±4.7
2nd DOL	84.0±9.7	84.2±9.1	85.4±7.3
3rd DOL	81.7±11.7	83.6±8.7	82.6±9.7
4th DOL	80.8±12.3	79.9±11.6	83.1±11.3
5th DOL	78.0±15.4	77.7±12-0	79.5±12.9
6th DOL	76.2±13.7	75.2±14.7	77.6±12.1
7th DOL	73.8±15.3	72.8±18.8	75.8±14.7
14th DOL	66.4±20.1	67.1±18.7	65.1±22.1
21st DOL	59.8±20.5	61.9±22.0	61.2±19.8
28th DOL	54.7±23.2	59.4±22.3	60.1±22.8
31st week of PMA	58.3±24.8	65.2±24.7	61.7±23.3
34th week of PMA	49.3±25.5	55.1±20.9	55.7±20.4
36th weeks of PMA	42.3±23.6	47.8±20.3	52.1±20.8*
P	<0.001	<0.001	<0.001

*P=0.036 vs. ICC
DOL: day of life PMA: postmenstrual age

Table S3. Fetal hemoglobin (HbF) levels (g/dL) measured between 1 and 7 days of life, at 14, 21, and 28 day of life and at 31, 34, and 36 weeks of postmenstrual age in infants who had resuscitation with immediate cord clamping (ICC), delayed cord clamping (DCC) or umbilical cord milking (UCM). Mean+SD.

	**ICC (n=120)**	**DCC (n=30)**	**UCM (n=31)**
1st DOL	14.0+3.1	14.6+3.5	15.1+2.7
2nd DOL	14.0+3.3	14.4+3.5	15.0+2.8
3rd DOL	13.1+3.4	13.9+3.5	13.6+3.0
4th DOL	12.6+3.6	13.0+3.9	13.7+3.1
5th DOL	12.0+3.6	12.8+4.3	12.9+3.5
6th DOL	11.6+3.3	12.1+4.0	12.4+3.4
7th DOL	11.2+3.4	11.7+4.9	12.0+3.9
14th DOL	8.9+3.7	9.5+3.9	9.7+4.1
21st DOL	7.5+3.4	8.3+4.2	7.9+3.2
28th DOL	6.3+3.1	7.2+3.6	7.1+3.0
31st week of PMA	7.5+4.1	8.3+3.8	8.3+4.5
34th week of PMA	5.2+3.2	5.6+3.2	5.8+2.6
36th weeks of PMA	4.2+2.4	4.8+2.0	5.4+2.4*
P	<0.001	<0.001	<0.001

*P=0.014 vs. ICC
DOL: day of life PMA: postmenstrual age
